# Cracked tooth syndrome: a diagnostic dilemma- a mini review

**DOI:** 10.3389/froh.2025.1572665

**Published:** 2025-06-24

**Authors:** Shreya Raj, Ankita Singh

**Affiliations:** ^1^ Department of Conservative Dentistry and Endodontics, DA Pandu Memorial RV Dental College, Affiliated with the Rajiv Gandhi University of Health Sciences, Bangalore, Karnataka, India; ^2^ Department of Conservative Dentistry and Endodontics, Manipal College of Dental Sciences Mangalore, Manipal Academy of Higher Education, Manipal, Karnataka, India

**Keywords:** cracked tooth, diagnosis, fracture, treatment plan, cracked tooth syndrome (CTS)

## Abstract

**Background:**

Cracked tooth syndrome is one of the five types of longitudinal fracture. It has been described as an incomplete fracture progressing from the vital tooth crown and progressing subgingivally, usually in a mesio-distal direction, involving dentin and often the dental pulp. Though termed as a syndrome it doesn't present with a set of classical symptoms. Hence, its diagnosis has always been arduous. This review paper summarises the current strategies in diagnosing a cracked tooth. This paper is an attempt to draw standardized protocols for diagnosing cracked tooth.

**Objective:**

The objective of this paper is to delve deeper into cracked tooth syndrome, thereby examining and simplifying each method to elucidate better its representation in clinics, such as history, examination, imaging etc. for an easier diagnosis.

**Clinical significance and relevance:**

Cracked tooth syndrome is a prevalent problem in dental practice. However, its ambiguous symptoms make a definitive diagnosis difficult, leading to a delay or failure to give appropriate therapy. Thus, it is imperative for a clinician to know about the various methods to correctly diagnose and provide apt and early treatment for cracked teeth.

## 1 Introduction

**Figure 1:**
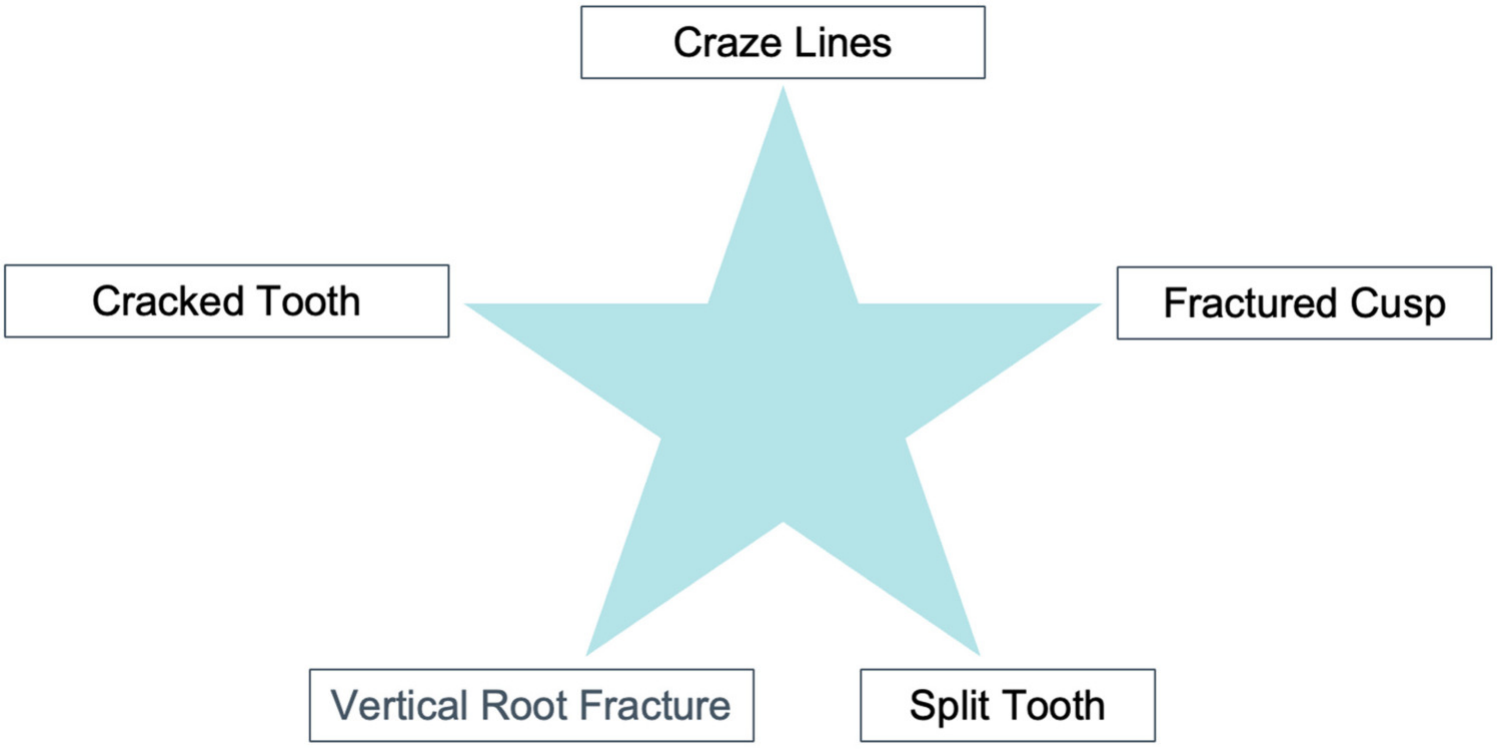
American association of endodontists classification of cracked teeth..

According to Dorland's Medical Dictionary, a syndrome presents as “a set of symptoms which occur together” (1). The term “cracked tooth syndrome” (CTS) was first described by Cameron (2, 3). It is one of the five types of Longitudinal fractures, described as an incomplete fracture initiated from the vital tooth crown and extending subgingivally, usually directed in a mesiodistal direction, involving dentin and often the dental pulp (Figure 1) (2–5).

A cracked tooth generally includes one or both of the marginal ridges. This fracture line is usually restricted to the crown of the tooth, although it can extend into the proximal surface of the root. These teeth could be at a higher risk of cuspal fracture, leading to more devastating consequences.

**Figure 2:**
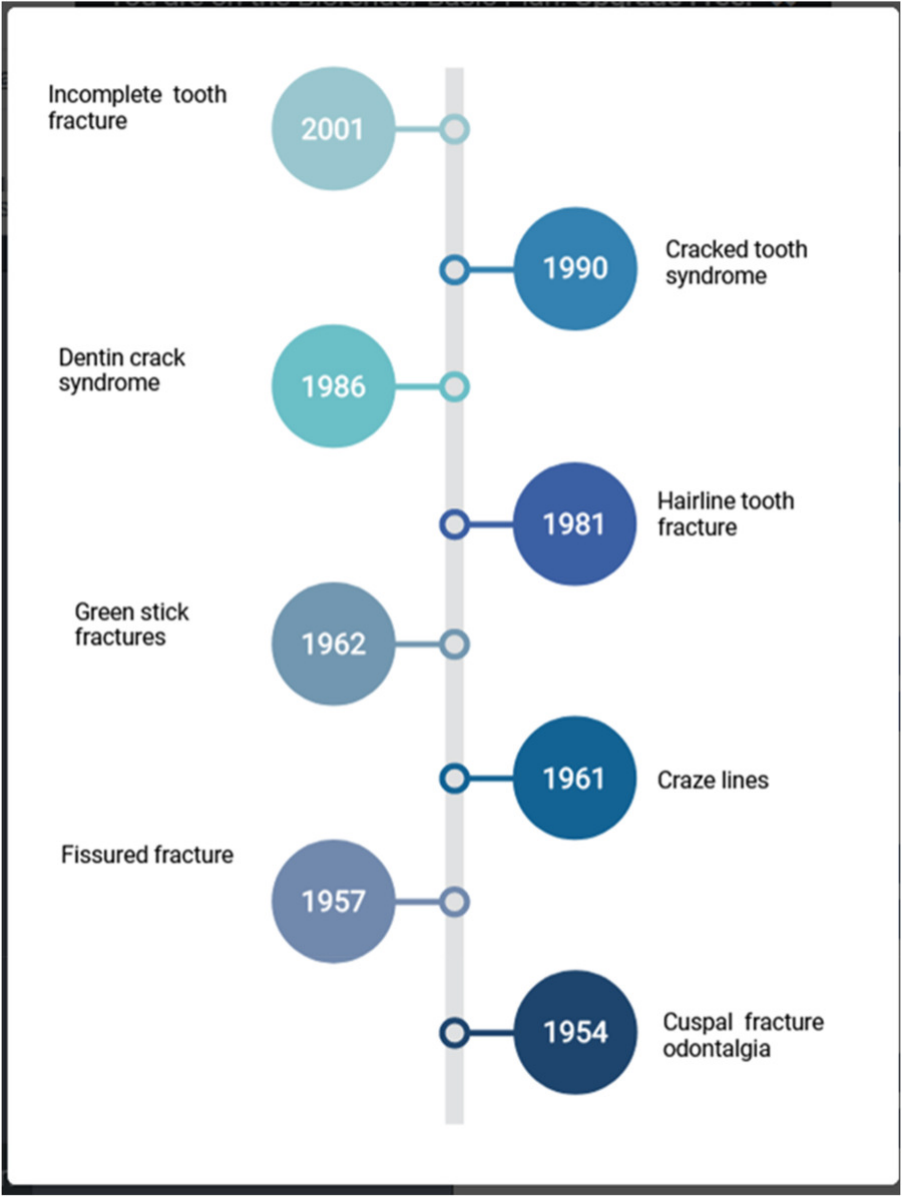
Changing terms over the years for cracked tooth syndrome.

Various terminologies have been proposed for describing cracks in the teeth (Figure 2) (6–11).

This has led to certain dubiety while attempting to describe clinicians' clinical presentation of cracked teeth (12).

**Figure 3:**
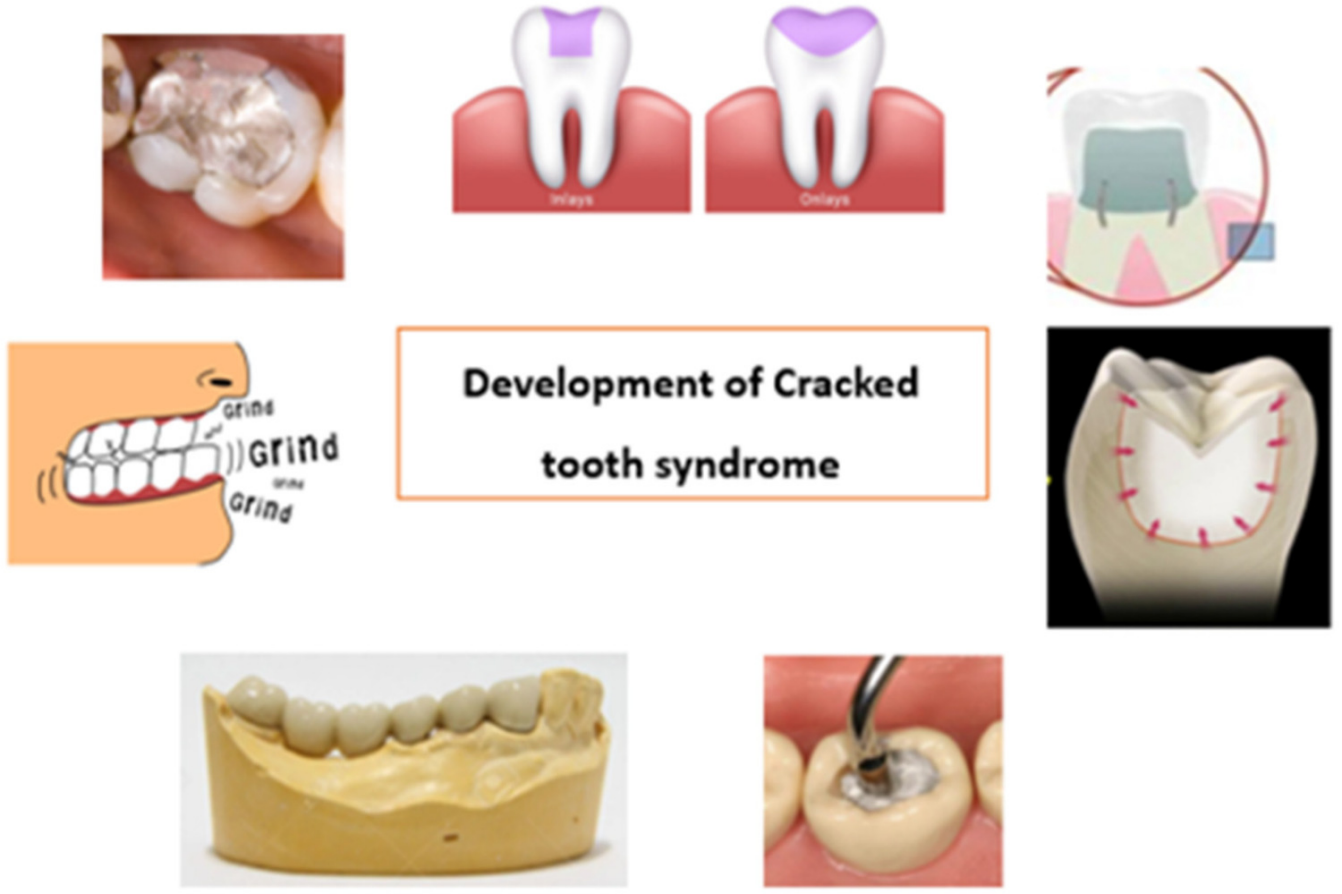
Causes of cracked tooth: (1) heavily restored teeth, (2) inlay/onlay (3) Complex amalgam restorations like pin restorations (4) large forces during restorative procedures like condensation (5) presence of long span bridges (6) parafunctional habits.

Various factors, including excessive teeth grinding, trauma, and aging, cause the condition. Signs and symptoms vary depending on the severity of the fracture but usually include pain and sensitivity to cold or hot foods and liquids (Figure 3). Diagnosis of CTS is often challenging due to its subtle nature and requires a multidisciplinary approach. This literature review will discuss the signs and symptoms of CTS and the diagnostic methods used to identify the condition accurately.

## 2 Methodology

A literature search was carried out on PubMed, MEDLINE (via Ovid), Embase (via Ovid), Scopus, and Web of Science databases for studies published up to March 25th 2025 using a combination of pre-specified “free-text” terms (keywords) and “subject headings”. The Google Scholar search engine was also used to ensure the comprehensiveness of the search and to identify any gray literature. The search process was also supplemented by manual searching in relevant dental journals and reference lists to identify studies missed by our electronic search.

### 2.1 Signs and symptoms

The cracked tooth syndrome is characterized by a plethora of symptoms that do not follow a clear or consistent pattern. Symptoms differ depending on whether the pulp is healthy, inflammatory, necrotic, or root canal treated (13).

The signs and symptoms of CTS are highly variable, depending on the severity of the fracture. The most common symptoms include:

• Location: Mandibular first molar is the most commonly affected teeth, followed by maxillary premolar, maxillary molar, and mandibular premolar. Mandibular first molars, being the first permanent teeth to erupt, are highly prone to dental caries and restorative treatments, making them more vulnerable to fractures. Additionally, the “wedging effect” from the prominent mesio-palatal cusp of maxillary first molars may further contribute to their susceptibility to fractures (14).
• Pain: Dull vague pain to characteristic “Rebound pain”. Rebound pain is pain experienced on release of pressure upon intake of fibrous food. This is caused by the fractured cusp flexing and repositioning (or “recoiling”), which activates the nerve fibers in dentine tubules from the odontoblastic layer, as well as from the hydrodynamic fluid movement inside the tubules (15).
• Sensitivity to cold or hot foods and beverages.
• Feeling something stuck in the mouth.

Other signs may include a decrease in masticatory function, sensitivity when biting down, and swelling of the gums.

## 3 Diagnostic methods

**Figure 4:**
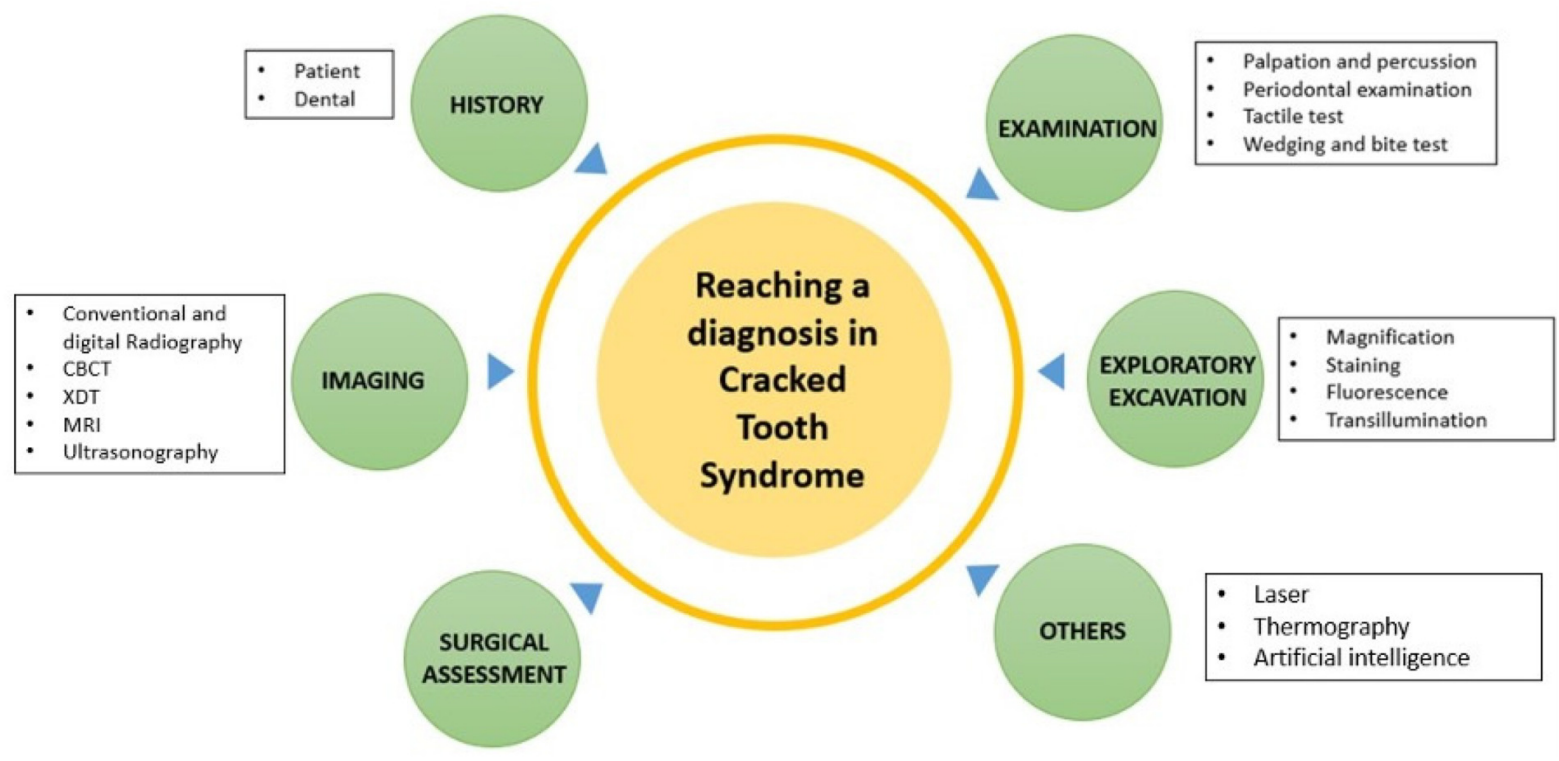
Tools in diagnosing cracked tooth.

Various diagnostic methods for CTS are used in dentistry, including visual inspection of the fractured tooth, palpation and percussion tests, radiography, endodontic probes, and transillumination (Figure 4) (16–18).

Visual inspection is the most common diagnostic tool for determining whether a tooth has been fractured. The clinician should inspect the tooth for discoloration, roughness, and sensitivity to percussion. Palpation and percussion tests also detect tenderness or pain in the fractured area.

Radiography is important for diagnosing CTS and differentiating between a cracked tooth and other conditions such as caries or periodontal disease (18). The radiograph should show a fracture line with a visible crown, root, and enamel-dentin junction.

Endodontic probes can also be used to diagnose CTS. The probe should be inserted into the gingival sulcus and then moved in a mesiodistal direction. If there is an obstruction, this indicates the presence of a crack. Transillumination is another technique used to diagnose CTS, which involves shining a light onto the fractured tooth. If a fracture line is visible, then this is an indication of CTS.

### 3.1 Subjective examination

#### 3.1.1 Patient history

Patients may fail to localize the problematic tooth and may complain of sensitivity (2, 4, 15). Discomfort while chewing is also one of the common complaints, which is characterized by acute pain on mastication (pressure or release) and sharp, brief pain with cold (14). Pain may range from mild in early stages to very severe spontaneous pain consistent with irreversible pulpitis, necrosis, or apical periodontitis (17). Patient gives a history of accidentally biting a hard object corresponding to the sudden onset of pain. History of damaging habits, such as clenching or grinding the teeth or chewing on ice, pens, hard candy, or other objects, may be reported. A history of a previous incident of a cracked tooth can help reach a diagnosis.

#### 3.1.2 Dental history

The patient may report the underlying cause of the pain and discomfort to have been misdiagnosed. A history of multiple occlusal modifications, with only temporarily relieved symptoms, or screening by several practitioners without a definitive diagnosis, all of which repeatedly indicated no cause for the pain or discomfort.

### 3.2 Objective visual examination

An additional oral examination reveals enlarged jaw muscles, which might indicate that the person grinds their teeth excessively hard regularly. By carefully analyzing the teeth in a dry condition, craze lines or darker fissures can be observed. The deeper the stains in the crack, the longer the fracture has been there. Other signs of a broken tooth include cracked restorations or unusual gaps between restorations and tooth structure. Wear facets, Cusp-fossae relationships, and a history of teeth clenching or grinding may enhance the probability of fractures.

### 3.3 Clinical examination

#### 3.3.1 Tactile examination

The tactile examination is done with the aid of an explorer. On the tooth's surface, a sharp explorer's tip would “catch” in a fracture when the explorer is moved (19).

#### 3.3.2 Palpation

Palpation may be ineffective in determining the presence of a crack. Palpating the gingiva surrounding the tooth, on the other hand, may indicate probable dehiscence or fenestration.

#### 3.3.3 Percussion

Percussion helps in determining whether a crack originating from the crown exists. Angular percussion (as opposed to straight vertical percussion) is very advantageous in diagnosis because it can split the fracture line, stimulate the periodontal ligament fibers, or cause fluid to move into the dentinal tubules, which can cause discomfort. Normally, opposite-direction percussion causes no symptoms.

#### 3.3.4 Wedging forces

When wedging forces are applied to a tooth suspected of having crack tooth syndrome, the tooth splits iatrogenically or causes discomfort. It may exacerbate the fracture line, which makes the tooth more prone to a later split. Pressure is applied on the opposing wall to separate the segments, and if no movement is noticed, the tooth is classified as a cracked tooth. A split tooth occurs when the segments separate.

#### 3.3.5 Bite tests

The bite test is intended to imitate the pathognomonic discomfort associated with cracked tooth syndrome that is felt during biting or shortly after the biting pressure is released. Also called “rebound pain” (2, 20). It can be replicated and this pain helps in the identification of a cracked tooth (21). This is caused by the fractured cusp flexing and repositioning (or “recoiling”), which activates the nerve fibers in dentine tubules from the odontoblastic layer, as well as from the hydrodynamic fluid movement inside the tubules (15). However, it is not a confirmatory test.

The bite test can be performed using a wooden toothpick, orange wood sticks, cotton wool rolls, abrasive rubber wheels such as Burlew wheels, or the head of a number 10 round bur in the handle of a cellophane tape (22). Two commercially available instruments are Fractfinder (Denbur, Oak Brook, IL, USA) and Tooth Slooth II (Professional Results Inc., Laguna Niguel, CA, USA). Tooth Slooth is composed of a little plastic biting block in the shape of a pyramid (20 × 10 mm) attached to a handle. We can put the pyramid's tip on each tooth's cusp suspected of having a fracture. The patient is instructed to bite with selective pressure on one cusp. Ehrman et al. suggested adopting this approach since it is more sensitive than wood sticks (20).

#### 3.3.6 Vitality testing

Establishing pulp vitality is critical in diagnosing the pulpal status, but more is needed to aid in crack identification. On the other hand, a crack that continues into the pulp may enable bacterial contamination, affecting the pulp's condition. Periapical and pulp testing findings are similarly varied. The pulp is normally responsive (vital) but can also be non-responsive (necrosis).

#### 3.3.7 Periodontal examination

Periodontal probing may reveal the approximate depth and severity of the fracture. Probing the full perimeter of the tooth in small increments may reveal a narrow, isolated periodontal probing defect, which is indicative of a crack. Subgingival fractures, on the other hand, do not always result in a probing defect. As a result, the absence of thorough probing does not rule out a broken tooth. The presence of deep probing is concerning and implies a worse prognosis. Yang et al. assessed the pulp status in cracked teeth based on periodontal probing depth (PPD) and discovered that cracked teeth with a PPD greater than 4 mm were more likely to have pulp necrosis (21, 23).

### 3.4 Imaging

#### 3.4.1 Radiographic examination

**Figure 5:**
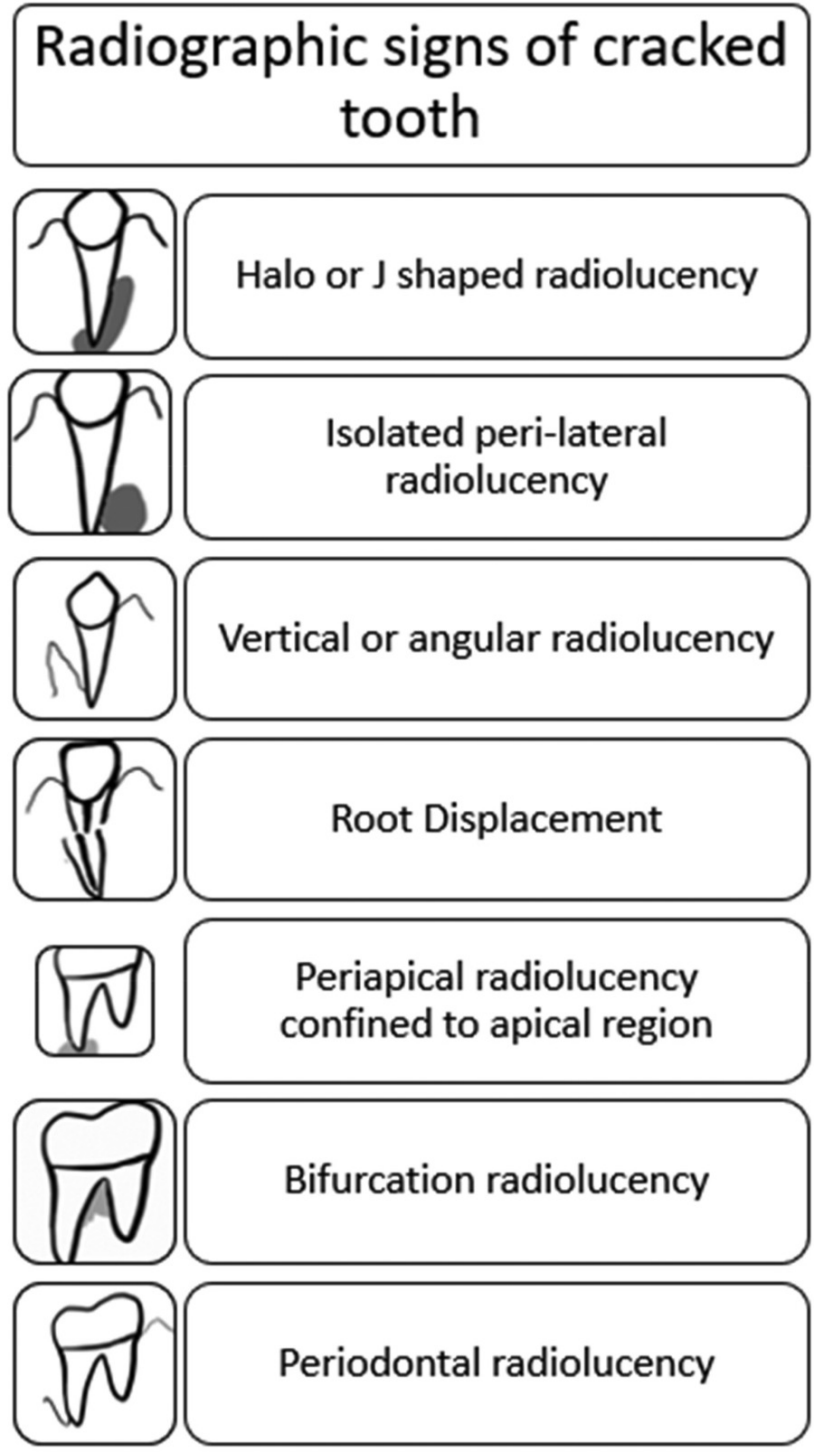
Radiographic signs of cracks and fractures in tooth.

Seldom are cracks diagnosed through radiographs. However, at later stages, when radiographic evidence of bone defects develops, a crack can be suspected. Taking periapical radiographs from many angles and bitewing radiographs enhances the likelihood of detecting a crack-induced defect early in its development (Figure 5). Thus, newer approaches, such as cone-beam micro-computerized tomography, are more useful in confirming a diagnosis.

Christoph Jud et al. recently proposed another 3-D imaging method in which they employed x-ray dark-field tomography (XDT) to identify dental microcracks (24, 25).

#### 3.4.2 Optical coherence tomography (OCT)

Optical coherence tomography (OCT) is an optical diagnostic tool based on interferometry. It can offer extensive information on tooth cracks, such as micro-fractures. It is a non-invasive method of diagnosing cracked-tooth syndrome. Sang-Hee Lee et al. effectively showed OCT as an additional approach for broken tooth diagnosis (24).

Sweep source OCT (SS-OCT) is a more sensitive method than previous OCT systems. It obtains subsurface cross-sectional images with the micron-level resolution, which aids in diagnosing cracks.

#### 3.4.3 MRI

The non-invasive and non-ionizing viewing of soft tissues using MRI has shown to be an optimal choice. In their study, Djaudat Idiyatullin et al. demonstrated the ability of SWIFT (Sweep Imaging with Fourier Transformation) MRI to detect cracks as small as 20 micrometers (µm), which is ten times smaller than the imaging voxel size (26).

#### 3.4.4 Ultrasonography

Without the associated risks with ionizing radiation, ultrasound can penetrate hard tissue, including radiopaque restorations (27). *In vitro* and *in vivo* Cracked tooth syndrome were successfully identified via ultrasonic systems by Culjat et al. and Sun et al., respectively (28).

### 3.5 Exploratory excavation

#### 3.5.1 Restoration removal exploratory excavation

Restorations often obscure the cracked tooth. Removal of restorations aids in the visual inspection of the cavity. Special attention is given to weaker areas like mesial and distal marginal ridges. Magnification, staining, transillumination, and application of wedging forces are useful aids in this process.

#### 3.5.2 Staining

To highlight fracture lines on the external tooth surface, in the cavity following restoration removal, or on a surgically exposed root, Iodine, Gentian Violet, or methylene blue dyes can be employed (29).

#### 3.5.3 Magnification

A level magnification of 14×–18× is recommended for the best assessment of enamel fractures. Additionally, it enables us to capture photographs for documentation and record keeping.

#### 3.5.4 Transillumination

Transillumination directly applies a fibreoptic transilluminator or other analogous light sources to the tooth surface (such as a fiber optic handpiece without water or a curing lamp). Transillumination illuminates the fracture from the area of the tooth where the light first penetrates. The fracture line reflects or blocks the light while it passes through structurally sound teeth. Clinically it is the cracked tooth's “night vs. day” appearance (30). Transillumination is particularly beneficial when performed after restorations are removed and are better when used with magnification (22).

#### 3.5.5 Fluorescence

Quantitative light-induced fluorescence (QLF) devices can diagnose tooth fractures. Crack lines get contaminated over time by oral biofilms and metabolites such as porphyrin and are seen as red fluorescent lines on QLF images (28, 29). Using QLF on extracted teeth, Jun et al. tried to determine the depth of enamel cracks non-destructively (22). Studies revealed that QLF images are more successful than radiographic and traditional visual examinations in identifying dental caries (Occlusal) and cracked teeth (31). Sung-Ae Son et al. showed the potential utility of QLF in detecting and characterizing tooth cracks, including their position and depth (32).

### 3.6 Surgical assessment

Surgical exploration can only be used to visually evaluate the root surface for the existence of a fracture if a crack is highly suspected and cannot be verified by any other method. As neighbouring teeth limit visibility in buccolingual cracks, they are easily identified after surgery.

#### 3.6.1 Others

##### 3.6.1.1 Laser

Lasers are used in dentistry for various purposes, especially as diagnostic and therapeutic aids. In a recent study, Ashita Sapra et al. demonstrated the ability of an 810-nm diode laser at 1W continuous wave to detect early signs of tooth crack (33).

##### 3.6.1.2 Thermography

This non-invasive imaging and visual inspection approach employ bio-thermal patterns. Matsushita-Tokugawa et al. discovered that Vibrothermography (VibroIR) might be a viable approach for identifying dentinal root microcracks by using friction heat generated by ultrasonic vibration (34).

##### 3.6.1.3 Artificial intelligence (AI)

Computers imitate intelligent behavior with minimum human interaction, referred to as artificial intelligence (AI). Treatments or image-based detection algorithms have been created to provide an error-free diagnosis. Fukuda et al. demonstrated that the CNN learning model could recognize VRFs (Vertical Root Fractures) on panoramic photos and operate as a CAD tool (35).

Three approaches to AI have been listed. The first is the CNN-based crack detection (Convolutional neural network) approach, where image classification, object detection, and semantic segmentation are thoroughly discussed. To be more specific, image classification-based algorithms (Alexnet) handle the crack detection issue as if it were a binary classification problem. However, its efficiency is slightly limited. Another technique is Object detection-based methods (YOLO, Faster R-CNN), where they immediately give information about the position and size of the targets of interest with a tagged bounding box in the picture. And finally, the third one is Pixel-level crack segmentation algorithms (Unet, Segnet, CrackSeg), a promising technique for fracture identification since they extract accurate information and more specific properties such as crack route, position, length, width, and density. However, technical challenges with AI-based detection may persist, such as high computing costs, issues selecting appropriate parameters, and the generation or pre-processing of training data sets (36–38).

##### 3.6.1.4 Management and prognosis

Formulating treatment plan for cracked tooth depends upon the extent of fracture line and the pulpal status (34, 39, 40).

The management of a cracked tooth largely depends on factors such as the location, orientation, extent, and severity of the crack. Cracks that are superficial are typically identified early, making them easier to treat. These minor fractures are commonly addressed with simple restorative procedures, like fillings or crowns.

However, when the crack extends deeper and involves the dental pulp, more extensive treatment such as root canal therapy followed by crown placement is necessary to preserve the tooth.

In severe cases, where the crack reaches below the gum line and into the tooth root, repair becomes unfeasible. Such situations usually necessitate tooth extraction, followed by replacement options like dental implants or bridges.

Clark and Caughman categorized the prognosis of cracked teeth into four levels: excellent, good, poor, and hopeless (41, 42).

• **Excellent prognosis**: Involves fractures within the dentin that angle from the cusp line angles toward the cemento-enamel junction, or slight subgingival extensions, or horizontal fractures of a cusp that do not affect the pulp.
• **Good prognosis**: Applies to vertical fractures confined to the crown that run mesiodistally into the dentin without reaching the pulp.
• **Poor prognosis**: Describes cracks that penetrate both the dentin and pulp but remain restricted to the crown.
• **Hopeless prognosis**: Indicates fractures that travel through the pulp and extend into the root structure, making tooth preservation unlikely.

## 4 Conclusion

Oral radiographs and cross-sectional images are the most useful methods for diagnosing cracked teeth. While MRI and ultrasonography have been used to identify the crack, they do not show the lesion's exact location. Laser, thermography, and surgical assessment are viable options, but they may need to provide more information to diagnose CTS accurately. AI-based techniques are a promising approach for crack detection, and they have the potential to provide more precise results than more traditional methods. However, AI-based approaches are still in development and will require further research before they can be applied to clinical practice. A combination of diagnostic tools is needed to diagnose CTS and provide effective treatment accurately.
